# Drug Testing for Newborn Exposure to Illicit Substances in Pregnancy: Pitfalls and Pearls

**DOI:** 10.1155/2011/951616

**Published:** 2011-07-17

**Authors:** Karen J. Farst, Jimmie L. Valentine, R. Whit Hall

**Affiliations:** ^1^Section for Children at Risk, Department of Pediatrics, University of Arkansas for Medical Sciences, 1 Children's Way, Slot 512-24A, Little Rock, AR 72202, USA; ^2^Section for Pharmacology and Toxicology, Department of Pediatrics, University of Arkansas for Medical Sciences, Little Rock, AR 72202, USA; ^3^Section for Neonatology, Department of Pediatrics, University of Arkansas for Medical Sciences, 4301 West Markham, Slot 512-B, Little Rock, AR 72205, USA

## Abstract

Estimates of the prevalence of drug usage during pregnancy vary by region and survey tool used. Clinicians providing care to newborns should be equipped to recognize a newborn who has been exposed to illicit drugs during pregnancy by the effects the exposure might cause at the time of delivery and/or by drug testing of the newborn. The purpose of this paper is to provide an overview of the literature and assess the clinical role of drug testing in the newborn. Accurate recognition of a newborn whose mother has used illicit drugs in pregnancy cannot only impact decisions for healthcare in the nursery around the time of delivery, but can also provide a key opportunity to assess the mother for needed services. While drug use in pregnancy is not an independent predictor of the mother's ability to provide a safe and nurturing environment for her newborn, other issues that often cooccur in the life of a mother with a substance abuse disorder raise concerns for the safety of the discharge environment and should be assessed. Healthcare providers in these roles should advocate for unbiased and effective treatment services for affected families.

## 1. Introduction

Estimates of illicit drug use in pregnancy vary widely. Approximately 5–10% of women self-report the use of illicit drugs in pregnancy [1–3], while universal testing for illicit drugs in high-risk populations results in a significantly higher prevalence (10–40%) of usage than through self-reporting [2, 3]. There is a wide range of use varying from infrequent recreational use to high levels of use with physiologic addiction. Importantly, other substances that can have deleterious effects on the mother and infants health (such as nicotine and alcohol) are often used concurrently with illicit drugs [1].

Identification of newborns exposed to illicit drugs in pregnancy cannot only alert the practitioner to problems one might encounter in the delivery room and nursery, but can also serve as an opportunity to recognize and assess families with substance abuse disorders which can pose risks to the newborn after hospital discharge. However, since self-reports of illicit drug use are often inaccurate and universal drug testing is neither practical for the clinician nor recommended by the American Academy of Pediatrics [4], every facility that provides care for newborns should establish their own testing protocol including establishing unbiased guidelines to identify those to be tested. Policies should be in place allowing for confirmation of test results that have been performed by screening methods which provide only presumptive results.

## 2. Possible Effects on Neonates due to Illicit Drug Use in Pregnancy

The short- and long-term adverse effects encountered by newborns exposed to illicit drugs in pregnancy can be difficult to accurately assess. *In utero* exposure to alcohol and nicotine has established potentials for negative effects on the newborn such as impairments in growth and later cognition [5]. While these substances are often used in conjunction with illicit drugs, they are rarely included in newborn screening or reporting policies [6]. As a result, studies examining the health effects of newborns exposed to illicit drugs in pregnancy can be confounded by the presence of other nonillicit substances whose presence can be difficult to control for in study design (especially if relying on self-reported usage). *In utero* exposure to alcohol and nicotine are the premier confounders. Also, effects attributed to illicit substance exposure during pregnancy may be confounded by the problems associated with substance abuse disorders such as poor nutrition, overall health status, and attendance at prenatal visits [7–9].

Table 1 provides a summary of possible adverse effects associated with exposure to the most commonly encountered illicit drugs (stimulants, cannabinoids, opiates/opioids, hallucinogens, and sedatives). While cocaine and methamphetamine both behave pharmacologically as stimulants (increased arousal, vasoconstriction, elevated heart rate, and blood pressure), much of the information about long-term effects in this class is derived from cohort studies on cocaine-exposed children. While there has been a longitudinal cohort study of children exposed to amphetamines *in utero* [26], long-term studies on children exposed to specifically methamphetamine are underway, but it is not yet known if there will be significant differences in long-term outcome. Inappropriate use of prescription pain medications (narcotics) and benzodiazepines are included as illicit drug usage [34].

Beyond the possible short- and long-term health effects, concern for the welfare and safety of newborns exposed to illicit drugs in pregnancy exists due to the cooccurring problems that many women with substance abuse disorders struggle with including undiagnosed/undertreated mental health issues, intergenerational addiction disorders within the family support system, and involvement in relationships with interpersonal violence [35–38]. The Adverse Childhood Experiences study group has shown that as the frequency of interpersonal violence increases in a child's home, so does the risk of becoming a victim of child abuse [39].

All newborns exposed to illicit drugs during pregnancy will not have adverse short- or long-term health effects, and the identification of a mother with a substance abuse disorder does not automatically infer the child will become a victim of abuse or neglect [40, 41]. The adequacy of the home environment is a strong factor in neurodevelopmental outcome [21, 23, 42] further highlighting the need to use identification of a newborn exposed to illicit drugs in pregnancy as an opportunity to be aware of problems that may manifest in the delivery room or nursery and assess the safety of the newborn's home environment to be along with the psychosocial situation of the family for needed supportive services [15].

## 3. Drug Testing in Newborns

In 2003, the United States Congress amended the Child Abuse Prevention and Treatment Act (CAPTA) by passing the *Keeping Children and Families Safe Act*. With this amendment, lawmakers conditioned a state's receipt of federal CAPTA funds on the establishment of procedures by the state to develop a plan of safe care when newborns exposed to illicit substances during pregnancy are reported by healthcare providers [43]. However, the Act leaves the decision on who should be tested to the healthcare provider. To avoid bias in testing towards newborns of women from poverty or minority backgrounds where substance abuse is sometimes assumed to be more of a problem, objective protocols for recognition of which newborns should be tested can be implemented [44–46]. The guideline from the authors' institution which was compiled from a previously published evidenced-based approach that identified maternal and newborn factors associated with illicit drug usage [43] and subsequently vetted with perinatal staff at the authors' institution is available in Table 2. The authors provide their guidelines and discussion and are not making a recommendation for adoption of what has been established at their institution as a universal standard.

Each healthcare facility should develop its own policy to address issues of consent in newborn drug testing. The intent of the test must be clearly defined. Testing for the purpose of guiding healthcare and followup after discharge may be covered on the general consent to treatment for the facility [47], whereas in the United States, testing for illicit substances in the absence of medical indications may be discriminatory and violate the patient's civil rights [48].

The healthcare provider has the responsibility to differentiate between screening and confirmatory drug testing results. This is especially true in cases in which a newborn has tested positive for an illicit drug and the mother has not admitted to usage. The potential for false positive testing by immunoassay screening should be acknowledged [49] and investigated further by ordering a direct identification, confirmation method such as gas chromatography-mass spectroscopy [44, 50]. The rate of false-positive immunoassay screening is particularly crucial with amphetamines and benzodiazepines [49].

Testing in newborns can be performed on urine, blood, meconium, hair, or umbilical cord blood or tissue samples. Immunoassay screening of urine and blood provide the most rapid results with urine usually preferred due to availability through noninvasive bag specimen collection. Drugs will clear rapidly from urine making false negative results possible when there is a delay in collection [8, 51, 52]. A laboratory's use of workplace standards for drug detection as opposed to lowest detectable limits can also lead to false negative screening results [44].

Meconium formation begins in 2nd trimester, and positive results typically reflect exposure in the last month or longer prior to delivery [44, 52]. Tests of meconium will more accurately identify a history of drug use rather than immediate drug use and are often more accurate than urine due to collection issues [3, 51]. First time drug usage just before delivery may result in a false negative meconium as the drug may not have had time for deposition. Therefore, urine testing may still be needed to cover the possible time periods of exposure prior to delivery. Results may not be available for several days after collection as meconium specimens that screen positive for drugs are typically confirmed by a direct identification method in a reference laboratory that performs such testing. While meconium results offer a wider window of exposure and more routine usage of confirmatory methods [53], it is not possible to clearly distinguish when in the last several weeks-months exposure occurred, and specimen collection can be difficult in newborns who have passed meconium *in utero* prior to delivery and in those who are very small/critically ill.

Neonatal hair growth begins in the third trimester [44, 52]. While not all newborns will have sufficient hair growth to allow for adequate specimen collection, hair drug testing may be helpful if meconium is not available due to transition to neonatal stool or clinical condition of the baby [52, 54]. Testing of the umbilical cord for *in utero* drug exposure is an alternative to meconium collection [55], but it is difficult to know how far back into pregnancy exposure would produce a positive test.

Clinicians in the nursery may be asked if it is reasonable that second hand smoke inhalation by the mother resulted in a positive newborn drug test. Passive exposure to heavy amounts of second-hand marijuana or crack cocaine smoke can result in a positive drug test in an exposed adult, but low levels of second-hand smoke exposure do not typically result in positive drug tests [56, 57]. If a mother is in an environment with others using drugs to the point that it is causing the mother and her newborn to test positive from passive exposure, the same concerns about home stability and cooccurring psychosocial risk factors should be communicated to personnel assessing the mother's situation since the newborn would be exposed to the same environment at discharge.

Confirmatory drug testing results may report either the parent drug and/or its metabolites. Therefore, the clinician should be familiar with basic drug metabolism of commonly abused drugs in order to account for exposure to certain parent compounds by the metabolites being detected during testing instead of the parent drug. In the stimulant class of drugs, methamphetamine is metabolized to amphetamine by the liver, but prescription amphetamine compounds will not metabolize to methamphetamine. Cocaine can metabolize to benzoylecgonine, norcocaine, ecgonine methyl ester (methylecgonine from crack), and if coingested with alcohol, cocaethylene [58]. Clinicians with questions about the consistency of clinical history with drug test results should consider consultation with a scientist from the reference laboratory that performed the confirmatory testing for the clinician's facility.

**Figure 1:**
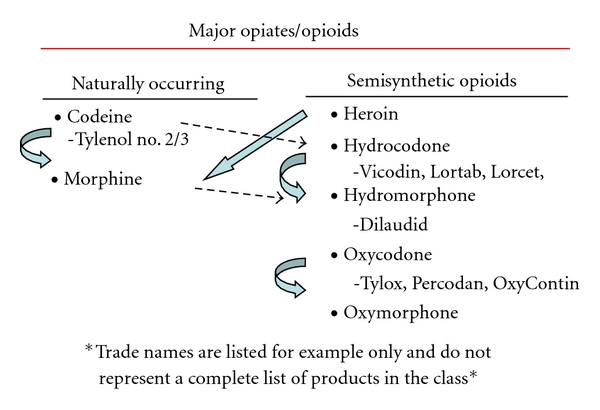
High morphine can show up with some hydromorphone, but generally hydro will break to hydro and oxy to oxy. Codeine can go to morphine and hydrocodone (not a metabolite of other opiates). Heroin breaks down to morphine and 6MAM. Codones can break to morhpones but not backwards. Hydrocodone can go to hydrocodol (= dihydrocodeine) and hydromorphone. Hydromorphone can go to hydromorphol (same for oxy but separate).

**Figure 2:**
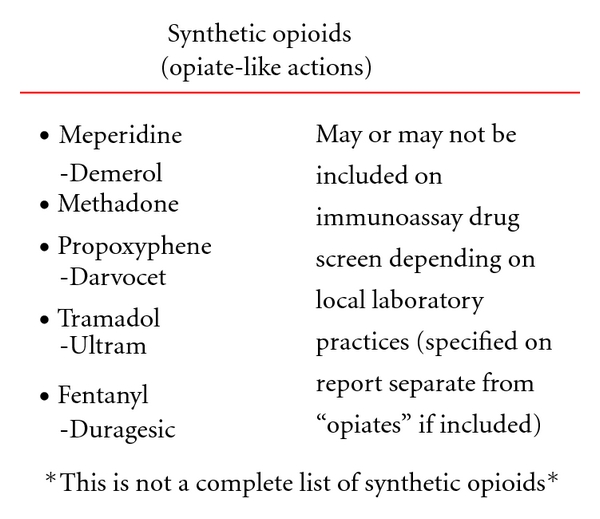


The opiate/opioid class of medications can be one of the most complex in regards to interpreting drug testing results [59]. These medications may be used legitimately for medical management of labor and delivery pain in the mother, neonatal pain after delivery, chronic medical conditions in the mother, and in addiction rehabilitation programs. Positive opiate results (morphine) can also be observed due to dietary intake of poppy seed containing foods although confirmation and quantitation of morphine will generally reveal urinary levels less than 800 ng/mL. However, they are also one of the most commonly inappropriately used/abused classes of prescription medications. Consultation with clinical toxicology experts is recommended to fully explore the interpretation of positive opiate results.  Figure 1 shows the division of this group of medications into primary opiates, semisynthetic opioids, and synthetic opioids with listing of common metabolites. It is important for the clinician in the nursery to understand that the synthetic opioids such as fentanyl or methadone would not be detected on routine toxicology screen for opiates. Specific testing would be required so their usage during labor and delivery or post delivery for pain management would not account for a positive screening test for opiates as is often assumed (see Figure 2).

## 4. Beyond the Nursery

As part of discharge planning, all newborns exposed to illicit drugs in pregnancy should have a primary care provider specifically designated to allow flow of information on risk status, referrals, and followup [60]. Caregivers with a substance abuse disorder are more likely to perceive care of a child as stressful and miss well-child visits [61]. Early intervention services should be considered because they can positively impact drug-exposed newborns at risk for developmental delay [62]. Nurse home visitation may be an appropriate referral in select cases [63]. Such programs may aid in reduction of subsequent encounters for ingestions, injuries, and maltreatment compared to controls [63, 64], or behavioral problems in children and in parental distress [65]. Perinatal healthcare providers should work collaboratively to educate state legislators that identification of drug use alone is not adequate to address the problems related to pregnant women with substance abuse disorders. States must develop a plan to assess families at risk by providing supportive services through their child welfare departments and include access to evidence-based substance abuse treatment programs. Providers should advocate for appropriate funding in child welfare budgets to ensure manageable case loads and staff training time. Prevention and family preservation instead of punishment will benefit the state in the long term by decreasing many of the other public health expenditures related to untreated substance abuse disorders.

## Figures and Tables

**Table 1:** Possible effects on newborns due to illicit drug use in pregnancy (not a complete list).

Drug	Possible effects on the newborn
*Stimulants:*	*Perinatal:*
Methamphetamine, Cocaine….	Low birth weight [10–12] CNS irritability/lability of state [13–15] —crying, jittery, sleep/wake alterations may have continued exposure through breastfeeding Neurodevelopmental alterations [16] Necrotizing enterocolitis [17] (Teratogenicity suggested by case studies but not confirmed by larger cohort or animal studies) [18]
	*Long term: * Modest but measurable longitudinal differences of *cocaine-exposed* infants in growth [19, 20], cognition [21], language [22], and impaired behavioral self-regulation [23, 24]. Other risk and protective factors can moderate outcome [23–25]. Longitudinal cohort of *amphetamine*-exposed infants showed school and behavioral problems (but environment impacts as well) [26]. Longitudinal *methamphetamine* studies are underway [27].

*Opiates/Opioids:*	*Perinatal:*
Heroin, morphine, codeine, oxycodone, hydrocodone, meperidine, fentanyl, (and others)	Low birth weight [8, 9] Neonatal Abstinence Syndrome (NAS) [15, 28] scoring system available: (i) CNS irritability (ii) Autonomic dysfunction (iii) Respiratory symptoms (iv) GI disturbances
	*Long term: * Longitudinal studies limited, problems with behavioral self-regulation reported [27].

*Cannabinoids*:	*Perinatal*:
Marijuana	Low birth weight with heavy exposure [29] Lability of state [15]
	*Long term*: Impulsivity [8] and effects on executive functioning later in life [8, 30]

*Hallucinogens*:	*Perinatal*:
PCP, MDMA, LSD	Low birth weight [7, 8, 13] CNS irritability [13] Neurodevelopmental alterations [31]
	*Long term*: Longitudinal studies not available

*Sedatives*:	*Perinatal:*
Benzodiazepines, barbiturates	Low birth weight [32] Respiratory depression, Hypotonia [33]
	*Long term*: Longitudinal studies not available

**Table 2:** Sample guideline for newborn drug testing.

Medical indications for NEWBORN drug testing for possible exposure to illicit drugs
*University of Arkansas for Medical Sciences, ANGELS Neonatal Guidelines *[46]
(1) History of maternal drug use or agitated/altered mental status in the mother
(2) No prenatal care
(3) Unexplained placental abruption
(4) Unexplained CNS complications in the newborn (seizures, intracranial hemorrhage)
(5) Symptoms of drug withdrawal in the newborn (tachypnea, hypertonicity, excessive stooling/secretions)
(6) Changes in behavioral state of the newborn (jittery, fussy, lethargic)
